# Factors related to and economic implications of inhospital death in German lung cancer patients - results of a Nationwide health insurance claims data based study

**DOI:** 10.1186/s12913-018-3599-3

**Published:** 2018-10-19

**Authors:** Karina Deckert, Julia Walter, Larissa Schwarzkopf

**Affiliations:** 1 Institute for Medical Information Processing, Biometry, and Epidemiology, Ludwig-Maximilians-University (LMU), Marchioninistr. 15, 81377 Munich, Germany; 2 Helmholtz Zentrum München GmbH, German Center for Environmental Health, Institute of Health Economics and Health Care Management, Member of Comprehensive Pneumology Center Munich (CPC-M), Member of German Center for Lung Research (DZL), Ingolstaedter Landstrasse 1, 85764 Neuherberg, Germany

**Keywords:** Lung carcinoma, Place of death, End of life care, Terminal care, Routine data, Palliative care, Health care supply structures

## Abstract

**Background:**

When patients die in a hospital their quality of life is lower than when they die at home or in a hospice. Despite efforts to improve palliative care supply structures, still about 60% of lung cancer patients die in a hospital. Studies have examined factors related to inhospital death in lung cancer patients, yet none used data of a representative German population, additionally including economic aspects. This study aimed to identify factors related to inhospital death in German lung cancer patients and analysed resulting costs.

**Methods:**

We analysed a dataset of health insurance claims of 17,478 lung cancer patients (incident 2009) with 3 year individual follow-up. We grouped patients into inhospital death and death elsewhere. Studied factors were indicators of healthcare utilization, palliative care, comorbidities and disease spread. We used logistic regression models with LASSO selection method to identify relevant factors. We compared all-cause healthcare expenditures for the last 30 days of life between both groups using generalized linear models with gamma distribution.

**Results:**

Twelve thousand four hundred fifty-seven patients died in the observation period, thereof 6965 (55.9%) in a hospital. The key factors for increased likelihood of inhospital death were receipt of inpatient palliative care (OR = 1.85), chemotherapeutic treatments in the last 30 days of life (OR = 1.61) and comorbid Congestive Heart Failure (OR = 1.21), and Renal Disease (OR = 1.19). In contrast, higher care level (OR = 0.16), nursing home residency (OR = 0.25) and receipt of outpatient palliative care (OR = 0.25) were associated with a reduced likelihood. All OR were significant (*p*-values< 0.05). Expenditures in the last 30 days of life were significantly higher for patients with inhospital death (€ 6852 vs. € 33,254, *p*-value< 0.0001).

**Conclusion:**

Findings suggest that factors associated with inhospital death often relate to previous contact with hospitals like prior hospitalizations, and treatment of the tumour or comorbidities. Additionally, factors associated with dying elsewhere relate to access to care settings which are more focused on palliation than hospitals. From these results, we can derive that implementing tools like palliative care into tumour-directed therapy might help patients make self-determined decisions about their place of death. This can possibly be achieved at reduced economic burden for SHIs.

**Electronic supplementary material:**

The online version of this article (10.1186/s12913-018-3599-3) contains supplementary material, which is available to authorized users.

## Background

In Germany, around 51% of all-cause and tumour-related deaths occur in a hospital setting, whereupon males (♂ 57% vs. ♀ 45%) and patients with tumours in respiratory organs (60% vs. 50%) are affected above average [1]. Previous research has found that irrespective of a cancer diagnosis, there is a preference for dying at home or in a hospice; dying in a hospital setting is favoured only by few (Gomes 2012, Gomes 2013, Pinzon 2011 and Higginson 2013) [2–5]. A palliative care setting may help patients express preferences about their place of death and their preferred treatment based on extensive information. Thus, patients in palliative care settings may more likely achieve their preferred end of life choices which supposed to improve their Quality of life (QoL) at the end of life. Although the number of inhospital deaths has declined in recent years, there is still an obvious discrepancy between the preferred and the actual place of death, especially in patients with respiratory tumours. Furthermore, this patient group is of high public health relevance, as respiratory tumours were the fourth leading cause of death in Germany in 2015 [6]. Two studies from the US and the UK found that, patients’ QoL at the end of life tends to be worse when they die in a hospital compared to when they die elsewhere, because they are more likely to experience physical and emotional distress and feel less at peace [7, 8]. Owing to different framework conditions, generalizing findings from one health care system to another is a sensitive issue. Of course, the results of the US-based study might not be fully replicable in the German setting, but since both systems are strongly “curatively” oriented and pay subordinate attention to palliative care, similar results can be expected for German populations. Additionally, inpatient care at the end of life generates higher expenditures than outpatient care as shown by Gaertner et al. (2013) [9] and Schwarzkopf et al. (2015) [10]. Thus, reducing the share of inhospital death in lung cancer patients is in the interest of both the patients concerned and health care service payers.

Costa (2014) published a systematic review on determinants influencing the place of death of terminally ill patients [11]. Interprofessional home palliative care and early referral to palliative services, inter alia, increased the number of home deaths. Based on these results, the German palliative care system has been improved, as Cremer-Schaeffer and Radbruch (2012) reported [12]. Since 2007, every German citizen has a legal claim to palliative home care. Furthermore, palliative care was implemented in educational programs for health professionals and the number of hospices increased overall. Consequently, Dasch et al. (2016) showed that the number of inhospital deaths in German lung cancer patients decreased between 2001 and 2011 (from 68,1% to 60,3% in men and from 70,7% to 49,6% in women) [1]. While trying to determine further factors influencing the place of death, Escobar Pinzón et al. (2011) found that nonworking relatives and a high care level are associated with home deaths in the German general population [4]. A study by Leak et al. (2013) describes characteristics of 104 lung cancer patients dying in emergency departments in North Carolina (USA) [13]. 71% of those patients died on their first visit and 65% of them were male. The most common chief complaint was respiratory distress. Considerably more patients (*n* = 143.627) were included in a UK nationwide study of O’Dowd et al. (2016) [14]. They identified sex, increasing age and social deprivation as factors associated with inpatient death.

To our knowledge, there is no representative nationwide study for a German population investigating multiple factors related to inhospital death as well as related to healthcare expenditures in German lung cancer patients as of yet.

Therefore, our study aimed to improve the evidence available by:

- Defining factors related to inhospital death based on statutory health insurance (SHI) claims data,
- using logistic regression models to identify factors associated with inpatient deaths, and
- comparing healthcare expenditures in the terminal phase between patients dying in a hospital and patients dying elsewhere.

## Methods

### Structure of the dataset

For our retrospective claims data analysis, we used nationwide insurance claims data by the AOK Research Institute (WIdO) [15] covering about 30% of the German population. According to the German Guidelines for Secondary Data Analysis [16] ethical approval is generally not required for this type of study. The German Reporting Standards for Secondary Data Analyses (STROSA) were considered in the preparation and implementation of this study [17].

The basic dataset contained anonymized data of 17,478 patients diagnosed with lung cancer in 2009, with patient-individual three-year follow-up (2009–2012). Details about the sample collection are described elsewhere [10]. Data included year and month of birth, sex, federal state of residence, care level (reflecting impairment in activities of daily living) and nursing home status over the course of the disease. Additionally, we had information on health care service utilization in the in- and outpatient setting, as well as on corresponding diagnosis codes (German International Classification of Diseases/ICD-10-GM) and medical procedures undertaken (OPS/German Version of the International Classification of Procedures in Medicine respectively GONR/outpatient billing codes).

Owing to data protection laws, date of death was only provided per month. For the purposes of this analyses we set date of death to the 15th of each month for any individual observed. Thus, there is an admitted imprecision of up to 2 weeks. To increase precision of date of death we subsequently checked the discharge status of hospital stays. Whenever death was documented here, we replaced the fictive data of death (15th of x) by the real date of death, which is date of discharge. Regarding the remainders, we searched for service provision after the 15th in the month of death. In case of service provision in the second half of the month, this date was assumed to be the date of death.

### Sample selection

**Fig. 1:**
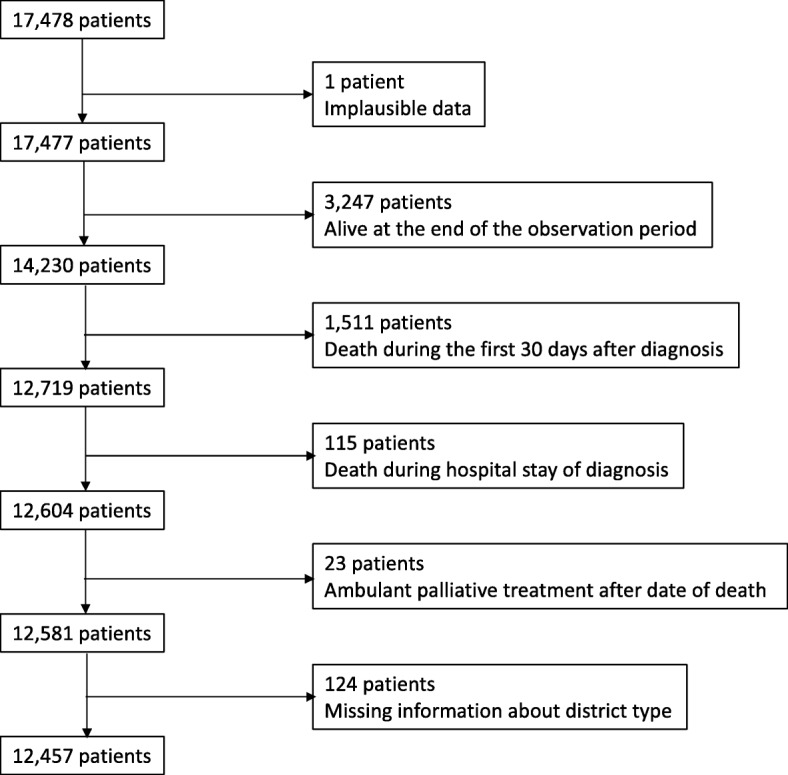
Patient flow diagram

As we compared place of death (inhospital or elsewhere) in our analysis, we excluded all patients who did not die within the individual three year follow-up period (*n* = 3247). One individual was excluded due to implausible data. To avoid a bias from patients with fulminant presentations and to focus on patients likely medically stable enough to have a choice regarding their place of death, we excluded patients who lived for less than 30 days after diagnosis (*n* = 1511). We further excluded any patients who died during the hospital stay of diagnosis (*n* = 115) and those with an implausible date of death, as they had claims for ambulant palliative treatments after date of death (*n* = 23). There are two possible reasons for claims of treatments after death. Firstly, outpatient palliative care also includes grief counselling for the relatives of the deceased. Secondly, as mentioned above date of death in our study can differ from actual date of death by a maximum of 2 weeks. Lastly, we excluded patients with unknown district type of residence at the time of diagnosis (*n* = 124). Our final study sample contained 12,457 patients (Fig. 1).

We assigned patients to the group inhospital death if the discharge status was ‘death’ in the last hospital visit. All other patients were assigned to the group of dying elsewhere.

### Factors related to inhospital death

We conducted a literature search in Pubmed in order to identify factors possibly associated with inhospital death. Search terms used included “place of death”, “end of life care”, “quality at the end of life”, and “factors influencing place of death” preferred for German lung cancer populations. We considered a potential association between inhospital death and following factors: age at time of death, gender, length of survival after diagnosis, nursing home residency, need for care, residence in Eastern vs. Western Germany, residence in rural vs. urban area, palliative care, previous healthcare utilization and treatment pattern, comorbidity burden and disease stage at diagnosis [4, 11, 13, 14].

Survival was measured as months survived after diagnosis. Nursing home residency (yes / no) and need for care defined as the patient’s care level refer to the last quarter before death. At the time of data collection, the German SHI system accounted for three care levels reflecting the patient’s capabilities to independently perform activities of daily living. The three levels of dependency were distinguished by how often assistance is needed and how long it takes a non-professional caregiver to help the dependent person. Higher care levels indicate increased need for assistance (i.e. greater physical or psychological impairment^1^) [18]. Living in Eastern or Western Germany as well as living in an urban or rural district was defined based on the ZIP code of the last documented residential address. We used the district types defined by the Federal Institute for Research on Building, Urban Affairs and Spatial Development for 2014, to classify the patients’ residential area as urban (district type 1 and 2) or rural (district type 3 and 4) [19].

Palliative care was included as a binary variable indicating whether palliative care measurements were administered at least once in an inpatient or outpatients setting. Information for this came from claims relating to palliative care codes (OPS for inpatient, GONR for outpatient palliative care).

We assessed previous healthcare utilization by calculating the number of days patients spent in a hospital, the number of outpatient hospital visits and of outpatient doctor visits (based on the number of days with a claim for a GONR) between diagnosis and death. To factor in collinearity with survival time we divided all those aspects by survival in months, resulting in utilization of healthcare by month survived.

To best possibly account for tumour stage at diagnosis –which is not documented within claims data– we used the type of tumour-directed therapy patients received and the location of metastases at baseline. We grouped patients using inpatient and outpatient ICD-10, OPS, billing and ATC codes into ‘no tumour-directed therapy’, ‘surgical resection’, ‘radiotherapy’, ‘chemotherapy’ and combinations of chemotherapy, radiotherapy and surgical resection. We identified patients with metastases at baseline using inpatient and verified outpatient ICD 10 codes in the quarters before and after the diagnosis of lung cancer and grouped them according to location; into thoracic, cerebral and bone metastases.

To reflect treatment intensity at the end of life, we reported chemotherapy given in the last 30 days before death as binary variable. This indicator of aggressive treatment has been used previously in end-of-life research [20].

To assess the patients’ comorbidity burden, we calculated the Charlson comorbidity index using the coding algorithm described by Sundararajan et al. (2004) [21] on all ICD-10 codes in claims in the 2 years prior to the diagnosis of lung cancer and included the distinct Charlson conditions as binary variables (comorbidity yes / no). As slight modifications from the initial algorithm, lung cancer was excluded from the condition ‘cancer’. Furthermore, we disregarded the condition ‘metastatic carcinoma’ to avoid a multicollinearity issue with the variable metastases location at baseline.

### Economic implications

To assess the economic implications of inhospital death we calculated expenditures for the health insurance company in the last 30 days before death. We compared total all-cause expenditures for hospitalizations, doctor visits and medications between patients with inhospital death to those who died elsewhere.

### Statistical methods

To investigate potential differences between lung cancer patients dying in a hospital and those dying elsewhere, we compared means, standard derivations (SD) medians and frequencies of basic variables like, gender, age and need for care. Categorical variables were compared using the χ^2^-test. Only age was approximately normally distributed (visual inspection) and compared using t-test (pooled test for equal variances). All other categorical variables were compared with Wilcoxon U-test for non-normal distributions. We compared survival trends of both groups via Kaplan-Meier curves and Log-Rank tests.

To examine the relationship between the related factors described above and inhospital death we used a multivariate logistic regression model and chose LASSO (“least absolute shrinkage and selection operator”) as the selection method [22]. LASSO selection combines some of the favourable properties of stepwise regression (ease of interpretation) and ridge regression (robustness) [22] while additionally performing better concerning multicollinearity [23, 24]. The first part of the LASSO loss function is equal to an ordinary least square regression, whereas the second part constrains the absolute value of the sum of the regression estimates by the parameter. It can be written as

$$ {L}^{Lasso}\left({\beta}_1,\dots, {\beta}_p\right)={\left\Vert Y-\sum \limits_{j=1}^p{X}_j{\beta}_j\right\Vert}^2+\lambda \sum \limits_{j=1}^p\left|{\beta}_j\right| $$

where L is the loss function, X is an n × p design matrix for predictors, Y is an n × 1 vector of responses, β is a p × 1 vector of regression coefficients, and λ ≥ 0 is the regularization parameter that controls the degree of shrinkage. Because the penalty term $\left(\lambda \sum \limits_{j=1}^p\left|{\beta}_j\right|\right)$ is based on the sum of the absolute values of the regression estimates, some estimates can be shrunken to exactly zero, which results in their exclusion from the model. That enables LASSO to be used for selection of predictor variables [25]. The LASSO loss function is not differentiable because of the unknown parameter λ. Thus, we used the method proposed by Nesterov (2013) to minimize the function while optimizing λ [26]. Age and sex were considered as pre-fixed covariates. To calculate odds ratios (ORs), 95%-confidence intervals (CIs) and *p*-values, we ran a logistic regression model with the covariates identified by LASSO selection. In this regard, it should be noted that all variables chosen by LASSO selection have an important influence on the outcome variable, irrespective of their statistical significance within the subsequent logistic regression model.

As expenditures did not show a normal Gaussian distribution using linear regression (OLS) was not possible. Therefore, we used a Generalized Linear Model (GLM) with gamma distribution and log link function. This kind of model is able to handle data that are right-skewed and eliminates heteroscedasticity [27]. The derivate of the parameter estimates from gamma regression represent the additive effect (in expenditures) that a change in this variable would cause. To interpret these results more easily, healthcare expenditures were reported as recycled predictions with confidence intervals and *p*-values. Recycled predictions are used to understand the marginal effect of independent variables on a dependent variable. They are obtained from the gamma regression model by averaging predicted scores, after fixing the value of one independent variable (either inhospital death, or death elsewhere), and using observed values on the remaining independent variables. The recycled predictions then provide adjusted means for both groups [28]. All recycled prediction models were adjusted by the factors identified as being associated with inhospital death earlier. The parameter estimates obtained from this gamma GLM are provided as Additional file 1. 

Confidence intervals and *p*-values of the adjusted means and difference were based on non-parametric bootstrapping (1000 bootstrap repetitions, percentile method). We used a significance level of 5% for all analyses.

To examine the robustness of the model we ran three sensitivity analyses (SA). For SA1 we extended the definition of inhospital death to include patients who were discharged from the hospital in the week before death and spent at least 2 days in the hospital in that week (additional 825 patients in inhospital death group). We chose the last week of life because it is defined as phase of death and we assumed that hospitalization in that final phase might correlate particularly strong with a decreased QoL [29]. Because our definition of inhospital death did not reflect whether a patient died on a general ward or on a palliative ward we ran SA2. Here, we reassigned 572 patients with inhospital death who had palliative treatments during the hospital stay of death to the group with death elsewhere. To avoid missing important information about patients with fulminant presentation (death during the first 30 days after diagnosis) we did not exclude these patients in SA 3 and performed the analysis with a sample of 13,090 patients.

All statistical analyses were performed using SAS version 9.4. Figures and tables were created with Microsoft Word, Power Point and Excel.

## Results

### Patient population and univariate analysis of factors related to inhospital death

Of the final study sample, 6965 individuals (55.9%) died in a hospital (Table 1). Patients with inhospital death were significantly younger than patients who died outside a hospital. Most of the patients were male (71.7%) with a significantly higher proportion of males in the inhospital death group. The residential setting was comparable in both groups regarding regional (Eastern vs. Western Germany) as well as structural (urban vs. rural) aspects. Nursing home residency was significantly less common in the inhospital death group, even though only few patients (7.7%) lived in a nursing home at all. Further, need for care was significantly less severe in those dying in a hospital. Mean Charlson comorbidity score did not differ significantly between the two groups. Patients dying in a hospital significantly more often had thoracic metastases (34.5% vs. 32.8%), other metastases locations did not differ.

**Table 1:** Description of the study sample

	Entire sample	Inhospital death	No Inhospital death	*p*-value^c^
n (%)	12,457 (100)	6965 (55.9)	5492 (44.1)	
Mean age at death (SD)	69.9 (10.0)	68.8 (10.0)	71.4 (9.9)	< 0.0001
Sex				
Male N (%)	8930 (71.7)	5070 (72.8)	3860 (70.3)	0.002
Survival				
Median survival in months	7	7	8	< 0.0001
Living in a nursing home ^a^ (%)	958 (7.7)	188 (2.7)	770 (14.0)	< 0.0001
Care level ^a^				
No care level N (%)	4958 (39.8)	3769 (54.1)	1189 (21.7)	< 0.0001
1 N (%)	2451 (19.7)	1324 (19.0)	1127 (20.5)	0.0351
2 N (%)	3416 (27.4)	1376 (19.8)	2040 (37.1)	< 0.0001
3 N (%)	1632 (13.1)	496 (7.1)	1136 (20.7)	< 0.0001
State				
Western Germany N (%)	9724 (78.1)	5478 (78.7)	4246 (77.3)	0.0732
Urban district ^b^ N (%)	7829 (62.9)	4380 (62.9)	3449 (62.8)	0.922
Mean Charlson Comorbidity Score (SD)	3.77 (2.65)	3.81 (2.66)	3.72 (2.63)	0.0777
Body regions with Metastases				
Cerebal N (%)	2271 (18.2)	1237 (17.8)	1034 (18.8)	0.1256
Bones N (%)	2845 (22.8)	1630 (23.4)	1215 (22.1)	0.0912
Thoracic N (%)	4207 (33.8)	2406 (34.5)	1801 (32.8)	0.0402

^a^ Data of the last quarter before death were used

^b^ Urban district is based on the district types defined by the Federal institute for Research on Building, Urban Affairs and Spatial Development for 2014. Urban district contains district type 1 and 2, the reference category rural district contains district type 3 and 4

^c^*p*-values were calculated with t-test for age at death, Wilcoxon U-test for Charlson Comorbidity Score and Chi^2^ test for sex, living in a nursing home, care level, state and urban district

**Fig. 2:**
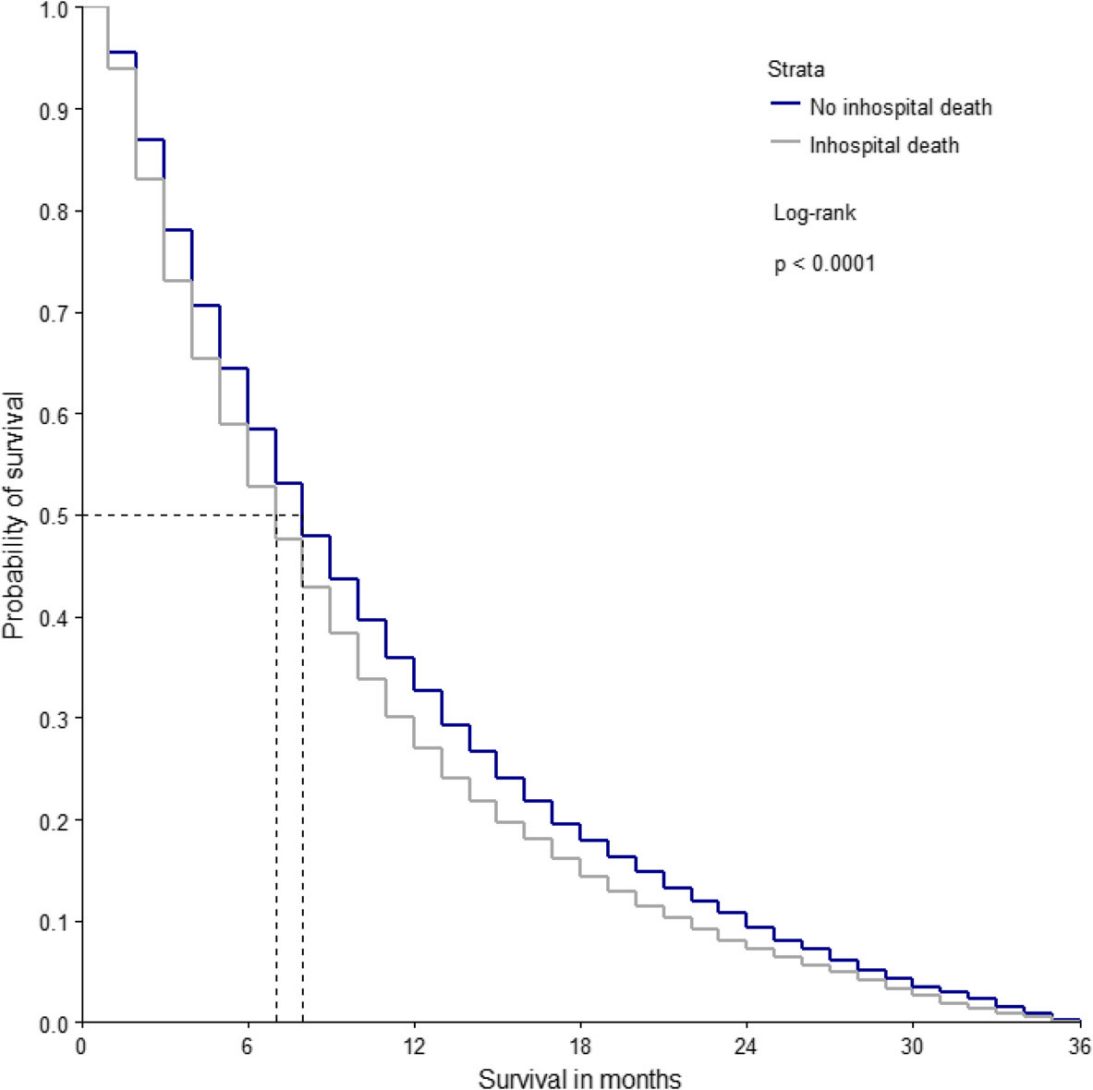
Kaplan-Meier-Curve to compare the survival of patients depending on place of death

Median survival was lower in patients dying in the hospital and the log-rank test showed significantly different survival between the two groups (Fig. 2).

Patients dying in a hospital were hospitalized significantly more often (10.9 vs. 8.4 days per month survived, *p*-value < 0.0001) and visited outpatient doctors significantly more frequently (3.1 vs. 2.8 visits per month survived, *p*-value < 0.0001).

The proportion of patients with inpatient palliative care was significantly higher in patients with inhospital death (18.0% vs. 13.4%, *p*-value < 0.0001) and the proportion of patients with outpatient palliative care was significantly higher in patients who died elsewhere (7.7% vs 1.6%, *p*-value < 0.0001).

The proportion of patients receiving chemotherapy in the last 30 days of life was significantly higher in the inhospital death group (30.0% vs. 16.7%, *p*-value < 0.0001). Overall, almost one-fifth of patients did not receive any tumour-directed therapy between diagnosis and death (*n* = 2.124, 17.1%). The proportion of patients not receiving cancer-directed therapy was significantly lower in the inhospital death group (12.9% vs 22.4%, *p*-value < 0.0001). Descriptive statistics of healthcare utilization, palliative care and therapy are displayed in Table 2.

**Table 2:** Descriptive statistics of healthcare utilization, palliative care and therapy

	Entire sample	Inhospital death	No Inhospital death	*p*-value^c^
n (%) Healthcare utilization ^a^	12,457 (100)	6965 (55.9)	5492 (44.1)	
Number of hospital days (SD)	9.8 (8.2)	10.9 (8.7)	8.4 (7.3)	< 0.0001
Number of outpatient doctor visits (SD)	3.0 (2.4)	3.1 (2.6)	2.8 (2.2)	< 0.0001
Number of outpatient hospital visits (SD)	0.01 (0.1)	0.01 (0.1)	0.02 (0.1)	0.0559
Palliative Care				
Inpatient palliative care N (%)	1994 (16.0)	1256 (18.0)	738 (13.4)	< 0.0001
Outpatient palliative care N (%)	2388 (4.3)	111 (1.6)	425 (7.7)	< 0.0001
Chemotherapy in last 30 days of life (%)	3009 (24.2)	2090 (30.0)	919 (16.7)	< 0.0001
Treatment ^b^				
No treatment N (%)	2124 (17.1)	896 (12.9)	1228 (22.4)	< 0.0001
Chemotherapy N (%)	2990 (24.0)	1745 (25.1)	1245 (22.7)	0.002
Radiotherapy N (%)	967 (7.8)	512 (7.4)	455 (8.3)	0.0532
Surgery N (%)	715 (5.7)	421 (6.0)	294 (5.4)	0.0996
Chemotherapy and Radiotherapy N (%)	3169 (25.4)	1867 (26.8)	1302 (23.7)	< 0.0001
Chemotherapy and Surgery N (%)	1016 (8.2)	632 (9.1)	384 (7.0)	< 0.0001
Radiotherapy and Surgery N (%)	301 (2.4)	158 (2.3)	143 (2.6)	0.2263
All three types N (%)	1175 (9.4)	734 (10.5)	441 (8.0)	< 0.0001

^a^ Healthcare utilization by month survived between diagnosis and death

^b^ Information if patients received this combination of treatments at any time of the observation period. Number or order of treatments was not considered

^c^*p*-values from Wilcoxon U-test for healthcare utilization, and from Chi^2^ test for treatment and palliative care

**Fig. 3:**
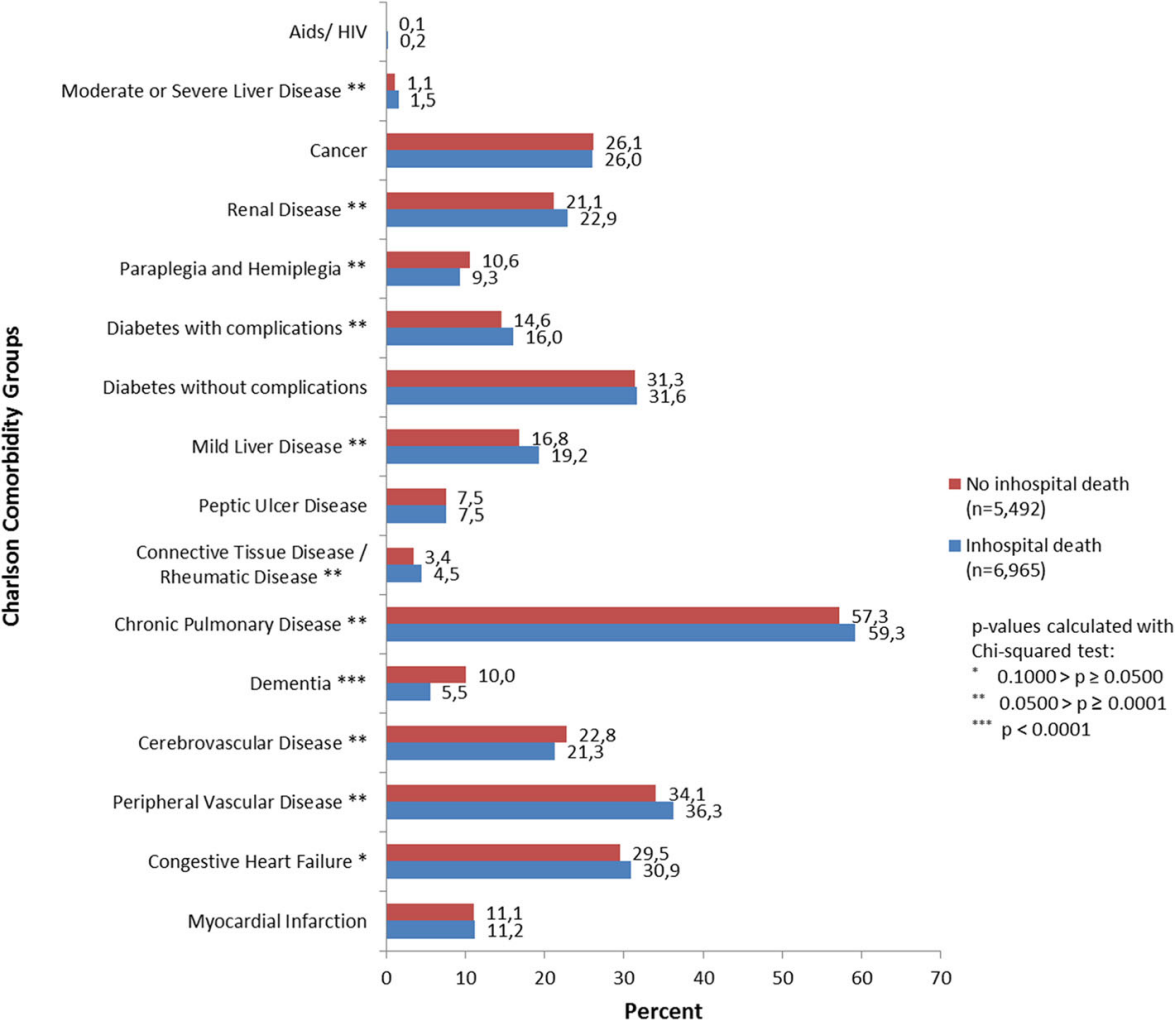
Prevalence of Charlson comorbidity groups depending on place of death

Regarding comorbidity burden, dementia was significantly less prevalent in the inpatient death group, whereas the prevalence of peripheral vascular disease, diabetes with complications and renal disease was significantly higher (Fig. 3).

### Multivariate analysis of factors related to inhospital death

The LASSO method selected the following as factors related to inpatient death: length of survival in months, nursing home residency, care level, number of hospital days, inpatient and outpatient palliative care, chemotherapy in the last 30 days of life, presence of congestive heart failure (CHF), and renal disease, as well as cerebral metastases (Table 3). Longer survival since diagnosis increased the odds of inhospital death. Nursing home residency, and increasing need for care reflected by higher care levels decreased the odds. Having spent more days in a hospital and receiving inpatient palliative care increased the likelihood for inhospital death whereas receiving outpatient palliative care resulted in a lower risk for inhospital death. If a patient received chemotherapy in the last 30 days of life, he was more likely to die in a hospital setting. Patients with CHF, or renal disease had an increased risk of inhospital death. Furthermore, patients with cerebral metastases had a decreased risk of inhospital death.

**Table 3:** Results of the logistic regression

	OR ^a, b^	95% CI ^a, c^	*p*-value
Age at death	0.985	0.98–0.989	< 0.0001
Sex			
Male vs female	0.95	0.87–1.04	0.2533
Survival in months	1.01	1.006–1.02	< 0.0001
Living in a nursing home	0.25	0.21–0.30	< 0.0001
Care level (reference = no care level)			
1	0.47	0.42–0.53	< 0.0001
2	0.26	0.24–0.29	< 0.0001
3	0.16	0.14–0.19	< 0.0001
Healthcare utilisation			
Number of hospital days ^d^	1.04	1.036–1.05	< 0.0001
Palliative Care			
Inpatient palliative care	1.85	1.66–2.08	< 0.0001
Outpatient palliative care	0.25	0.20–0.32	< 0.0001
Chemotherapy in last 30 days of life	1.61	1.46–1.77	< 0.0001
Charlson Comorbidities Groups			
Congestive Heart Failure	1.21	1.11–1.33	< 0.0001
Renal Disease	1.19	1.08–1.32	0.0006
Body regions with Metastases			
Cerebral metastases	0.86	0.77–0.96	0.0050

^a^ Values are rounded to two decimals except if they would be rounded to exactly 1 then they are rounded to three decimals

^b^ Odds ratio

^c^ 95% confidence interval

^d^ Healthcare utilization by month survived between diagnosis and death

### Multivariate analysis of expenditures

All-cause total costs were significantly higher for patients dying in a hospital (€ 6852 vs. € 3.254, *p*-value < 0.0001). Hospitalizations made up the biggest part of expenditures and were significantly higher in patients with inhospital death (€ 5895 vs. € 2321, *p*-value < 0.0001). Expenditures for doctor visits were significantly higher in patients who did not die in a hospital (€ 358 vs. € 245, *p*-value < 0.0001), as were expenditures for medications (€ 691 vs. € 585, *p*-value < 0.0001).

### Sensitivity analyses

The results of SA1 (Additional file 2) and the main analyses were similar, however the LASSO method did not result in an inclusion of renal disease as an important factor anymore. The results of SA2 (Additional file 2) and the main analyses differed in two aspects. First, chronic pulmonary disease (CPD) was selected as important factor additionally, increasing the odds of inhospital death. Second, inpatient palliative care now decreased the likelihood of inhospital death.

The model in SA3 was almost the same as in the main analysis, some effect sizes changed in decimal places only.

## Discussion

In our study, we sought to identify factors related to inhospital death in German lung cancer patients. We found that especially the patients’ age and frailty, as measured in the need for care and nursing home residency, were important factors related to not dying in a hospital. In addition, patients with cerebral metastases had a decreased risk of inhospital death. The utilization of outpatient palliative care also reduced the likelihood of inhospital death but receiving inpatient palliative care increased the likelihood of inhospital death. Furthermore, a higher number of days spent in a hospital, CHF and renal disease, as well as receiving chemotherapy in the last 30 days of life were associated with an increased risk of inpatient death.

Total healthcare expenditures were about twice the expenditures of dying elsewhere, with expenditures for hospitalizations predominantly responsible for these differences. Although expenditures for outpatient care and medications were higher in patients not dying in the hospital, the sum of these differences were negligible compared to the differences in inpatient expenditures.

Evidence, in which direction age affects inhospital death is inconclusive: Dasch et al. found a negative association for the general population in a North Rhine-Westphalia-based study (< 60 versus > 80 years: OR 1.41, *p*-value 0.001) [30], but O’Dowd et al. observed a positive association among lung cancer patients in the UK (> 85 year vs. 70–74 years OR 1.16; 95% CI 1.08–1.24) [14]. Finally, Escobar Pinzón et al. (2011) found no association at all between age and place of death in a population of Rhineland-Palatinate, however this study was not focused on lung cancer patients but referred to the general population [4]. We believe that our observation of negative association is plausible: Younger patients may be subject to more aggressive inpatient therapy. Therefore, their primary clinical relationship is rather with an oncologist than with a palliative care team. Moreover, they might have more social/familial obligations than older patients and death at home may not be desirable if they have a young family at home. Consequently, younger patients have an increased risk of inpatient death.

Longer survival after diagnosis increased the risk of inhospital death. This was a surprising result at first as we assumed that patients with shorter survival have less time to come to terms with impending death, and therefore to organize their final days in a preferred and comfortable place. However, if a patient is diagnosed in a late stage of the disease the focus of care is more likely to be on palliation and organizing end-of-life care rather than curative therapy. In line with that, patients with cerebral metastases at time of diagnosis died in the hospital less frequently.

In our study nursing home residency was associated with a reduced likelihood of dying in a hospital. This might be explained by the fact that continuous nursing care — as often required at end of life — is already available in this setting, whilst it can often not be guaranteed in an ambulatory setting. Even though dying in a nursing home or in a hospice is not the same as dying at home, most patients prefer those places to hospitals [2]. Relatives who are not able to arrange care of the patients at home due to financial or other reasons, thus should be supported more relating organization of accommodation in a hospice or a palliative care facility in order to increase the patient’s QoL during the last days. Wye et al. (2014) describe that arranging care outside the hospital is a very tough task for the relatives [31]. For this reason, the UK has implemented end of life care services, which provide nurses who support relatives with organizational services, as well as assistance with decision-making. These nurses are supposed to offer time for conversations with the patients and the relatives about death, as well as the practical aspects of caring for the dying. This concept works especially for cancer patients who are close to death and should be considered as a developing concept to further improve end of life care in Germany. Actually, at the time this study was conducted, there was study ongoing to evaluate establishing a system of ‘social care nurses’ in cancer therapy in Germany funded by the German Ministry of health.

Our study indicated that patients with lower care levels are more likely to die in a hospital. Escobar Pinzón et al. (2011) found similar results in his study (care level 3 vs no care level: OR (death elsewhere vs inhospital death) 4.95, *p*-value < 0.0001) [4]. The higher the care level the higher the need for support in various aspects of everyday life. Thus, comprehensively dealing with the organization of the required care has already taken place. If a home-care setting for a person with high nursing needs has been organized previously, it is far easier to enable the patients dying outside a hospital – as hospitalization is necessary less frequently, and relatives are able to handle complications outside a hospital (with assistance of professionals).

According to our study, CHF and renal diseases increased the probability of inhospital death. These comorbid conditions tend to require intense treatments and are associated with a high likelihood of complications. Managing these complications often requires further inpatient hospital care [32]. However, it should be examined whether it is possible to further train formal and informal care-givers to manage these conditions outside the hospital.

If a patient in our study received inpatient palliative care, he was more likely to die in a hospital setting. This finding was unsurprising as inpatient palliative care is administered in a hospital for example on a palliative care ward. Our SA 2 supports this assumption as in this analysis we regrouped patients from the inhospital death group, if they had an inpatient palliative care treatment in the hospital stay of death. Resulting in the effect of inpatient palliative care changing direction and now reducing the likelihood of inpatient death.

Given, that outpatient palliative care was per se provided on a low level among patients of the inhospital death group, a conclusive interpretation of these results is limited to some extent. By trend, our analyses support the study of Purdy et al. (2015), which reported that patients receiving outpatient palliative care for a longer duration were less likely to die in a hospital. Patients and relatives using palliative care have usually already confronted the fact of impending death. Most families feel overburdened in this situation. The providers of palliative care are in a better position to assist the family with decision-making and to help them with enabling the patient’s death in a preferred place – mainly because there is sufficient time and opportunity for such conversations. Furthermore, palliative care providers have a broad base of knowledge about the health care system as well as local supply structures. In addition, engaging with outpatient palliative care may define a population who are more determined to die at home. Purdy et al. (2015) [33] demonstrated that an end of life coordination centre that aims to help patients under palliative care be cared for in their preferred place, can reduce the number of inhospital deaths significantly (OR 0.33, *p*-value < 0.001).

In contrast to receiving palliative care, the administration of chemotherapy in the last 30 days of life was associated with an increased likelihood of inhospital death. Receiving chemotherapy shortly before death can be considered as an aggressive treatment and deemed undesirable by some clinicians [34, 35], even though it needs to be kept in mind that palliative care could to some extent also include chemo-therapeutic measures. That these patients more often die in a hospital could be explained by the close relationship between the patient and the oncologist who is often located in a hospital. Furthermore, death might be associated with the treatment itself while in the hospital.

Similar reasons could play into the finding that patients with a higher number of hospital days also have a higher likelihood of inpatient death. A higher number of hospitalizations may indicate a more aggressive or longer tumour-directed treatment. Therefore, patients have an established relationship with the hospital and its clinicians and associate them rather than a palliative care team with the primary point of contact.

Despite higher comorbidity burden, patients in the inhospital death group were less frail as measured in care level and nursing home residency. Therefore, we can deduct that it’s not the patients’ frailty that has an impact on the place of death as these patients are already cared for at diagnosis, but more importantly specific comorbidities. Therefore, these comorbidities are important aspects to consider when lung cancer is diagnosed.

As expected, we found that expenditures for inpatient care were around double in the inhospital death group. However importantly, we could see that the excess costs in the outpatient sector for patients who did not die in the hospital were almost negligible. From this, we can conclude that not only costs for hospitalizations are much higher, but also additional expenditures for outpatient care are extremely low.

Thus, extending outpatient palliative care services as it currently happens in Germany will probably create a win-win situation for patients and the SHI system, since the expected decline in expenditures for inpatient care would compensate for the additional expenses in the outpatient sector. Our results are supported by the studies of Faßbender (2005) [36] and Smith et al. (2012) [37] who found that palliative care might have a cost-cutting effect because of fewer therapeutic interventions not primarily aiming at improving QoL for terminally ill patients. Thereby the need for inpatient treatments might be reduced and patients remain in a stable condition while still receiving sufficient medical and non-medical care.

Most limitations in our study stem from the use of claims data, which are primarily documented by healthcare providers for billing purposes with health insurance funds. The exact place of death is not important for these parties and is therefore not documented. This is the reason that within this study we have only been able to distinguish between inhospital death and death elsewhere. We do not know if patients in the inhospital death group died on a palliative ward or on a normal ward, nor can we differentiate between various places of death outside a hospital such as a hospice or the family home. Owing to data protection laws, data contained only year and month of death for those who died in an outpatient setting. We used rules to define a more valid date of death but there are still possible differences to the actual date of death of up to 2 weeks for each individual. As O’Malley et al. (2005) described, the coding of ICD- and OPS-codes is a source of error in itself [38] which can e.g. arise out of unintentional and intentional coder errors, such as misspecification, unbundling, and up-coding. This should be considered when interpreting variables based on those codes like Charlson comorbidity groups. For historical reasons, the average income and the family structure of AOK-insured people may differ from the general German population. When there was no free choice of health insurance provider, the AOK as a Local Health Care Funds insured the general population (workers, retirees, family members) in contrast to company-provided health insurance funds, which for example insured predominantly persons with higher incomes and who tends to be healthier. Even now when all members of the population are in the position to choose their own health insurance provider, this structure is still visible. However, Jaunzeme et al. (2013) have shown that these differences are small enough to assume that the population of AOK-insured and herewith our results are representative for the German resident population [39].

Our study represents the first nationwide examination of factors possibly related to inhospital death in German lung cancer patients such as the patients’ care needs, comorbidity burden, lung cancer-related treatments, and regional aspects. More importantly, we could provide expenditures for inhospital death not only as total but also for different aspects of care and therefore provide evidence that investments in palliative care can nevertheless reduce expenditures in total. As an innovative methodological approach, we applied the LASSO selection method which combines the easy interpretability and robustness. LASSO selection has been shown to outperform other selection methods like stepwise selection [40]. Finally, our analyses are automatically based on a multicentre study, whereby biases resulting from specific documentation rules and approaches of single healthcare providers are avoided. Thus, we are convinced that these analyses create a sound starting point for conditions that need to be considered when trying to create an environment supporting terminally ill-patients to make a more self-determined decision on where to spend their last days of life.

## Conclusion

This study provides further evidence about factors related to inhospital death in German lung cancer patients. Findings suggest that factors associated with inhospital death often relate to previous contacts with hospitals. This includes prior hospitalizations, tumour-directed treatment and treatment of comorbidities. Additionally, factors associated with dying elsewhere relate to access to other care settings than hospitals where therapy is focused more on palliation. From these results we can implicate that an early or earlier integration of palliative care into tumour-directed therapy might be a useful tool in helping patients to make informed decisions in the last phase of their life, by using established relationships e.g. with the oncologist. Additionally, further expanding the palliative supply network, can be achieved while still reducing costs for SHIs.

## Additional files

Additional file 1: Parameter estimates from gamma regression costs. The table shows the parameter estimates from the gamma regression of costs in the last 30 days of life. Costs comprise total all-cause expenditures for hospitalizations, doctor visits and medications and are compared between patients with inhospital death to those who died elsewhere. (PDF 68 kb)

Additional file 2: Results of the logistic regression in SA 1 and SA 2. We ran the logistic regression with lasso selection method for SA 1 and SA 2 to proof the robustness of the main analysis. (DOCX 20 kb)

## Abbreviations

AOK: A Local Health Care Funds
CHF: Congestive heart failure
CI: Confidence level
CPD: Chronic pulmonary disease
MLD: Mild liver disease
OR: Odds ratio
PVD: peripheral vascular disease
QoL: quality of life
SA: Sensitivity analysis
SD: Standard deviation
SHI: Statutory health insurance
WIdO: AOK Research Institute

## Footnotes

1 Care level I: People who need assistance with personal hygiene, feeding or mobility for at least two activities from one or more areas at least once a day, and who additionally need help in the household several times a week for at least 90 min a day with 45 min attributable to basic care. Care level II: People who need assistance in at least two basic activities of daily living (ADLs) at least three times a day at various times and additional help in I ADLs several times a week for at least 3 h a day, with 2 h attributable to basic care. Care level III: People who need assistance in at least two ADLs around the clock and additional help in an IADL several times a week for at least 5 h per day, with 4 h attributable to basic care.
