# Supplementary material for: Factors related to and economic implications of inhospital death in German lung cancer patients - results of a Nationwide health insurance claims data based study
Source: BMC Health Serv Res. 2018 Oct 19;18:793. doi: 10.1186/s12913-018-3599-3 (PMC6194570; doi:10.1186/s12913-018-3599-3)
Supplement: Supplementary file 1 — Parameter estimates from gamma regression costs. The table shows the parameter estimates from the gamma regression of costs in the last 30 days of life. Costs comprise total all-cause expenditures for hospitalizations, doctor visits and medications and are compared between patients with inhospital death to those who died elsewhere. (PDF 68 kb) [file 12913_2018_3599_MOESM1_ESM.pdf]

	ß	95% CI [a, c]	p-value
**Inhospital death**	0,65	0.62-0.68	<.0001
**Age at death**	-0,01	-0.013- -0.099	<.0001
**Sex**			
Male vs female	0,07	0.03-0.10	<.0001
**Survival in months**	0,01	0.01-0.01	<.0001
**Living in a nursing home**	-0,18	-0.24- -0.12	<.0001
**Care level** (reference = no care level)			
1	-0,15	-0.20- -0.11	<.0001
2	-0,15	-0.19- -0.11	<.0001
3	-0,17	-0.21- -0.12	<.0001
**Medical consultations**			
Number of hospital days	0,03	0.03-0.03	<.0001
**Palliative Care**			
Inpatient palliative care	0,18	0.14-0.22	<.0001
Outpatient palliative care	0,03	-0.04-0.10	0,4497
**Chemotherapy in last 30 days of life**	0,21	0.17-0.24	<.0001
**Charlson Comorbidities Groups**			
Congestive Heart Failure	0,03	0.00-0.07	0,0554
Renal Disease	0,05	0.01-0.09	0,008
**Body regions with Metastases**			
Brain	0,01	-0.03-0.05	0,5515

[a] Values are rounded to two decimals except if they would be rounded to exactly 1 then they are rounded to three decimals

[c] 95% confidence interval
