# Supplementary material for: Factors related to and economic implications of inhospital death in German lung cancer patients - results of a Nationwide health insurance claims data based study
Source: BMC Health Serv Res. 2018 Oct 19;18:793. doi: 10.1186/s12913-018-3599-3 (PMC6194570; doi:10.1186/s12913-018-3599-3)
Supplement: Supplementary file 2 — Results of the logistic regression in SA 1 and SA 2. We ran the logistic regression with lasso selection method for SA 1 and SA 2 to proof the robustness of the main analysis. (DOCX 20 kb) [file 12913_2018_3599_MOESM2_ESM.docx]

Additional file 2

|  | **Regression model sensitivity analysis 1** | | | **Regression model sensitivity analysis 2** | | | |
| --- | --- | --- | --- | --- | --- | --- | --- |
|  | **OR ^a^** | **95% CI ^b^** | **p-value** | **OR** ^a^ | **95% CI** ^b^ | **p-value** |  |
| **Age at death** | 0.98 | 0.97-0.98 | <.0001 | 0.98 | 0.98-0.99 | <0.0001 |  |
| **Sex**  Male vs female | 0.97 | 0.89-1.06 | 0.4572 | 0.96 | 0.88-1.04 | 0.3231 |  |
| **Survival in months** | 0.99 | 0.99-1.00 | 0.0329 | 0.97 | 0.97-0.98 | <0.0001 |  |
| **Living in a nursing home** | 0.33 | 0.28-0.38 | <.0001 | 0.32 | 0.27-0.39 | <0.0001 |  |
| **Care level** (reference = no care level)  1  2  3 | 0.53  0.31  0.19 | 0.47-0.59  0.28-0.34  0.17-0.21 | <.0001  <.0001  <.0001 | 0.51  0.29  0.18 | 0.46-0.57  0.27-0.32  0.16-0.20 | <0.0001  <0.0001  <0.0001 |  |
| **State**  Western Germany | 0.82 | 0.75-0.91 | <.0001 |  |  |  |  |
| **Medical consultations**  Number of days in hospital  Number of outpatient medical consultations | 1.01  1.03 | 1.00-1.02  1.01-1.05 | 0.0038  0.0017 | 0.99  1.03 | 0.98-0.99  1.01-1.05 | <0.0001  0.0014 |  |
| **Palliative Care**  Number of days with inpatient palliative care  Number of days with outpatient palliative care | 0.99  0.93 | 0.98-1.00  0.91-0.96 | 0.1035  <.0001 | 0.96  0.91 | 0.95-0.97  0.88-0.94 | <0.0001  <0.0001 |  |
| **Treatment** (reference = no treatment)  Chemotherapy  Radiotherapy  Surgery  Chemotherapy and Radiotherapy  Chemotherapy and Surgery  Radiotherapy and Surgery  All three types |  |  |  | 1.48  1.55  1.54  1.72  1.84  1.62  2.03 | 1.30-1.69  1.31-1.83  1.28-1.87  1.50-1.98  1.55-2.20  1.24-2.12  1.71-2.43 | <0.0001  <0.0001  <0.0001  <0.0001  <0.0001  0.0004  <0.0001 |  |
| **Charlson Comorbidities Groups**  Congestive Heart Failure  Peripheral Vascular Disease  Dementia  Chronic Pulmonary Disease  Mild Liver Disease  Diabetes with complications  Renal Disease | 1.23  1.13  0.85  1.10  1.14  1.15 | 1.12-1.35  1.04-1.23  0.73-0.99  1.01-1.19  1.02-1.28  1.04-1.27 | <.0001  0.0056  0.0327  0.0219  0.0219  0.0080 | 1.28  1.12  0.97  1.06  1.09  1.16  1.22 | 1.17-1.40  1.03-1.22  0.83-1.13  0.98-1.15  0.99-1.21  1.03-1.29  1.11-1.35 | <0.0001  0.0068  0.6636  0.1287  0.0949  0.0111  <0.0001 |  |
| **Body regions with Metastases**  Lymph Nodes  Pleura  Others | 1.13  1.27  0.94 | 1.04-1.22  1.12-1.44  0.86-1.03 | 0.0030  0.0003  0.1842 | 1.02  0.89 | 0.95-1.11  0.82-0.98 | 0.5628  0.0125 |  |
| ^a^ Odds ratio.  ^b^ 95% confidence interval. | | | | | | | |
